# Evaluation of the Efficacy of Human Papillomavirus Screening Compared With Cytology Screening in Pregnant Women: Protocol for a Prospective Multicenter Trial

**DOI:** 10.2196/86397

**Published:** 2026-06-25

**Authors:** Risa Matsunaga, Jun Tamura, Kouji Yamamoto, Kotaro Senuki, Reo Tanoshima, Etsuko Miyagi, Taichi Mizushima

**Affiliations:** 1 Department of Obstetrics and Gynecology Yokohama City University Graduate School of Medicine Yokohama, Kanagawa Japan; 2 Department of Biostatistics Yokohama City University School of Medicine Yokohama, Kanagawa Japan; 3 YCU Center for Novel and Exploratory Clinical Trials Yokohama City University Hospital Yokohama, Kanagawa Japan; 4 Department of Health Data Science, Graduate School of Data Science Yokohama City University School of Medicine Yokohama, Kanagawa Japan

**Keywords:** uterine cervical neoplasms, mass screening, pregnancy, human papillomaviruses, cytology

## Abstract

**Background:**

Although the overall number of cervical cancer cases is declining worldwide, the incidence of this type of cancer is increasing in regions with low human papillomavirus (HPV) vaccination and cancer screening rates. In such regions, screening pregnant women can help improve the overall screening rate. Worldwide, cervical cancer screening is shifting from cervical cytology to HPV testing, which reduces the incidence of cervical cancer and, when negative, allows for longer screening intervals. Although HPV-based screening has been shown to be at least as effective as cytology screening for lesion detection in the general population, its performance during pregnancy remains poorly characterized due to the exclusion of pregnant women in previous clinical trials.

**Objective:**

The aim of this trial is to evaluate the effectiveness of an HPV testing protocol compared with that of a cervical cytology protocol for cervical cancer screening in pregnant women.

**Methods:**

This study is a multicenter, nonrandomized, single-arm comparative trial. In total, 5000 pregnant women aged 30 years and older in their first trimester will undergo both cervical cytology and HPV testing, with follow-up at 1 year. The primary end point is to assess the noninferiority of the HPV testing protocol compared with the cytology protocol for detecting cervical intraepithelial neoplasia grade 2 or higher, adenocarcinoma in situ, and invasive cervical cancer (CIN2+) in early pregnancy. In the cytology protocol, CIN2+ cases will be defined as those detected in cytology-positive participants. In the HPV protocol, CIN2+ cases will be defined as those detected in HPV-positive and cytology-positive participants. The primary end point analysis will use a one-sided 10% noninferiority test using the method by Tango, and with 5000 cases, the study can achieve a statistical power of at least 80%. Secondary end points will include an evaluation of the persistence of negative results post partum to determine the optimal screening interval in this population.

**Results:**

The trial was funded in April 2024 and has already started. Data collection commenced in September 2024 and is scheduled to be completed by December 2026. As of March 2026, a total of 1906 participants have been recruited. Data analysis is currently planned to begin in December 2027, and the study findings are expected to be published in 2028.

**Conclusions:**

The results of this trial will clarify whether the HPV testing protocol should be adopted as the primary cervical cancer screening method for pregnant women or whether cervical cytology should remain the standard of care.

**International Registered Report Identifier (IRRID):**

DERR1-10.2196/86397

## Introduction

More than 90% of cervical cancer cases are related to human papillomavirus (HPV), with carcinogenesis initiated via persistent infection with HPV [1]. The World Health Organization advocates for a cervical cancer elimination strategy with 3 key targets: a 90% HPV vaccination rate, a 70% cervical cancer screening rate, and a 90% treatment rate for precancerous and cancerous lesions [2].

Although the incidence of cervical cancer and mortality has been declining in countries that have consistently met these targets, their global incidence continues to increase, and considerable regional disparities remain [3]. In Japan, cervical cancer cases are on the rise [4], with approximately 11,000 new diagnoses and 2900 deaths annually. Cervical cytology–based screening is a well-established method that reduces mortality [5]. However, Japan’s screening uptake rate was only 43.6% in 2022 [6]. Prenatal visits provide a valuable opportunity for screening because more than 80% of pregnant women in Japan receive cervical cancer screening during these visits [7]. In some Southeast Asian countries, including India and Indonesia, the incidence of cervical cancer remains high while screening uptake is below 50% [8,9]. In such settings, incorporating screening into pregnancy care may improve participation.

**Figure 1:**
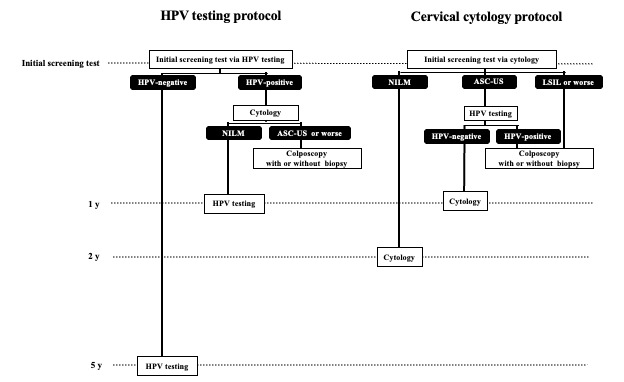
Current cervical cancer screening protocol in Japan. In the human papillomavirus (HPV) testing protocol, HPV-negative cases are scheduled for another HPV test in 5 years. HPV-positive cases undergo cervical cytology. For cases positive for HPV and atypical squamous cells of undetermined significance (ASC-US), colposcopy with or without cervical biopsy is performed immediately. Repeat HPV testing is scheduled within 1 year for HPV-positive and negative for an intraepithelial lesion or malignancy (NILM) cases. In the cervical cytology protocol, cases that are NILM are scheduled for another cervical cytology 2 years later. Cases with ASC-US undergo HPV testing, and cervical cytology is scheduled 1 year later for cases with ASC-US and who are HPV negative. For cases with ASC-US and who are HPV positive or for low-grade squamous intraepithelial lesion (LSIL) or worse cases, colposcopy and cervical biopsy are performed immediately.

HPV testing has also been shown to reduce the incidence of cervical cancer [10]. Compared with a negative cytology report, a negative HPV test is associated with a lower risk of developing precancerous lesions and permits extended screening intervals [11]. Accordingly, global cervical cancer screening strategies are gradually shifting from conventional cytology screening to HPV screening [10], and long-term effectiveness has also been demonstrated in real-world clinical practice [12]. In Japan, an HPV testing protocol has been available since 2024 at the discretion of individual municipalities. In the HPV testing protocol (Figure 1), HPV-positive participants undergo cervical cytology; those with atypical squamous cells of undetermined significance (ASC-US) or worse receive immediate colposcopy with or without biopsy, whereas those who are negative for an intraepithelial lesion or malignancy (NILM) undergo repeat HPV testing at 1 year. In the cytology protocol (Figure 1), participants with ASC-US undergo HPV testing; those with ASC-US and who are HPV positive or with low-grade squamous intraepithelial lesion (LSIL) or worse receive immediate colposcopy and biopsy, whereas those with ASC-US and who are HPV negative undergo repeat cytology at 1 year. Under Japan’s current HPV testing protocol (Figure 1), the screening interval for HPV-negative individuals can be extended from 2 to 5 years compared with the interval under the conventional cytology protocol [13].

Pregnant women have been excluded from most HPV screening trials. Therefore, the efficacy of HPV screening in this population has yet to be determined. The aim of this trial is to evaluate the effectiveness of the HPV testing protocol compared with that of the cervical cytology protocol for cervical cancer screening in pregnant women.

A noninferiority trial design was selected for this study. The number of cervical intraepithelial neoplasia (CIN) grades 2 and 3, adenocarcinoma in situ (AIS), and invasive cervical cancer (hereafter abbreviated as CIN2+) cases detected during pregnancy is expected to be higher under the cytology protocol than under the HPV testing protocol as the cytology protocol includes a broader population for confirmatory colposcopic evaluation, making a superiority trial design for HPV screening infeasible. Large-scale studies in nonpregnant women have demonstrated that HPV testing is comparable or superior to cytology in detecting CIN and invasive cervical cancer. In addition, HPV testing allows for the extension of the screening interval when negative; therefore, even if lesion detection rates are similar, the HPV testing protocol offers meaningful clinical advantages.

## Methods

### Study Aim and Design

**Figure 2:**
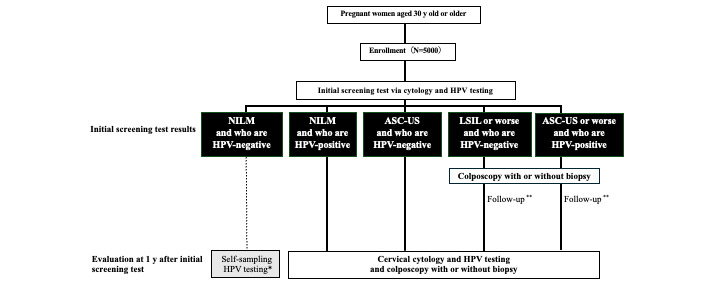
Follow-up protocol of the HOPER (Human Papillomavirus Testing for Pregnant Woman: Pregnant Women Health Initiative) trial. Low-grade squamous intraepithelial lesion (LSIL) or worse cases include participants with LSIL, atypical squamous cells cannot exclude a high-grade squamous intraepithelial lesion (HSIL; ASC-H), HSIL, squamous cell carcinoma (SCC), atypical glandular cells (AGC), adenocarcinoma in situ (AIS), adenocarcinoma, or other malignancies. Atypical squamous cells of undetermined significance (ASC-US) or worse cases include participants with ASC-US, LSIL, ASC-H, HSIL, AGC, SCC, AIS, ADCA, or other malignancies. The solid lines indicate procedures performed at all participating sites, whereas the dotted lines indicate procedures performed at selected sites only. *Self-collection human papillomavirus (HPV) testing will be conducted at 2 predefined facilities. **During pregnancy, cervical cytology and/or colposcopy with or without cervical biopsy is performed every 3 to 6 months for this subset of participants. NILM: negative for an intraepithelial lesion or malignancy.

This is a multicenter, nonrandomized, comparative trial designed to demonstrate the noninferiority of an HPV testing protocol compared with a cervical cytology protocol for detecting CIN2+ cases in pregnant women. In this study, all participants will undergo both HPV testing and cervical cytology using cervical swab specimens collected during early pregnancy (Figure 2). Confirmatory evaluation and follow-up will be performed to satisfy each protocol, and the expected number of CIN2+ cases detected under each protocol (Figure 1) will be estimated and compared.

Ethical and efficiency considerations informed the decision to use a nonrandomized trial design.

### Eligibility Criteria

This study includes only pregnant women aged 30 years or older, consistent with HPV testing protocols in Japan and many other countries given that HPV screening in women in their 20s may increase false-positive results [13]. Women at less than 17 weeks of gestation are eligible because cervical cancer screening is recommended in early pregnancy in Japan [14]. The exclusion criteria were as follows: women with no cervix, women who had been previously diagnosed with cervical cancer, women who were monitored by a health care provider for a diagnosis or suspicion of CIN, and other conditions considered unsuitable for inclusion by the physician.

### Primary End Point

The primary end point is the number of CIN2+ cases diagnosed immediately following cervical cancer screening in early pregnancy.

The difference in detecting CIN2+ cases between HPV testing and cervical cytology protocols is expected to be minimal [15]. Therefore, this trial was designed to establish noninferiority of the HPV testing protocol in detecting true positive cases of CIN2+ immediately after screening.

### Secondary End Points

The secondary end points are as follows:

- Comparison of the HPV testing protocol with the cervical cytology protocol for detection of CIN grades 1, 2, and 3; AIS; and invasive cervical cancer during early pregnancy and 1 year later
- HPV subtype positive rate; abnormal cervical cytology positive rate; and CIN detection rate during early pregnancy per subgroup, including age, history of past pregnancies, delivery status, marital status, HPV vaccination coverage, smoking history, and oral contraceptive use
- HPV subtype positive rate; abnormal cervical cytology positive rate; and CIN detection rate 1 year later per subgroup, including HPV subtype results in early pregnancy, cytology results in early pregnancy, age, pregnancy, delivery status, marital status, HPV vaccination coverage, smoking history, and oral contraceptive use
- CIN and cancer detection rate during pregnancy and 1 year later per subgroup, including HPV subtype, cytology finding, age, pregnancy, delivery status, marital status, HPV vaccination coverage, smoking history, and oral contraceptive use
- Comparison of cervical cytology results between this trial (Cytopick brush [Matsunami Glass Industries, Ltd] or liquid-based cytology) and conventional screening
- Comparison of HPV test results between self-sampling and physician sampling
- Self-sampling HPV positive rate among HPV-negative and cervical cytology–negative cases 1 year later
- Number of people eligible for colposcopy or biopsy via the HPV testing protocol and cervical cytology protocol
- Adverse events
- Comparison of the cumulative rate of lesion progression among HPV-positive pregnant women according to immunostaining results

### Baseline Screening and Colposcopy or Biopsy During Early Pregnancy

Pregnant women will be recruited from prenatal care facilities in Japan, including those affiliated with the Yokohama Clinical Research Network.

After obtaining informed consent, cervical swab specimens will be collected at less than 17 weeks of pregnancy using the BD SurePath test with the Cytopick brush for both cervical cytology and HPV testing using the BD Onclarity assay. The results of HPV testing will be classified as high-risk HPV negative or high-risk HPV positive if they are positive for any of the evaluated genotypes (HPV16; 18; 31; 45; 51; 52; 33 and 58; 35, 39, and 68; or 56, 59, and 66).

The results of cervical cytology will be classified as follows according to the Bethesda system [16]: NILM, ASC-US, LSIL, atypical squamous cells cannot exclude a high-grade squamous intraepithelial lesion (HSIL; ASC-H), HSIL, squamous cell carcinoma (SCC), atypical glandular cells (AGC), AIS, and adenocarcinoma. LSIL or worse results include participants with LSIL, ASC-H, HSIL, SCC, AGC, AIS, adenocarcinoma, or other malignancies. ASC-US or worse results include participants with ASC-US, LSIL, ASC-H, HSIL, AGC, SCC, AIS, adenocarcinoma, or other malignancies.

Participants with an abnormal cervical cytology result, except for those with ASC-US and who are HPV negative, will undergo colposcopy and, if necessary, a cervical tissue biopsy.

CIN is classified according to the World Health Organization criteria as LSIL (CIN grade 1), HSIL (CIN grade 2), and HSIL (CIN grade 3) [17]. The primary end point, CIN2+ cases, is defined based on histological diagnosis of biopsy specimens by pathologists at each participating site using the same criteria. For participants requiring diagnostic evaluation, colposcopy is performed promptly, and the timing of this procedure is recorded in the case report form, allowing it to be clearly distinguished from the 1-year follow-up.

### Follow-Up During Pregnancy and After Birth

The study follow-up scheme is shown in Figure 2. Participants with positive cervical cytology results (excluding those with ASC-US and who are HPV negative) will undergo cervical cytology and/or colposcopy with or without a cervical biopsy every 3 to 6 months and at 1 year after the baseline screening. Participants with ASC-US and who are HPV negative and those who are NILM and HPV positive at baseline screening will undergo cytology, HPV testing, and colposcopy with or without cervical biopsy at 1 year after the baseline screening. Participants who are NILM and HPV negative at baseline screening will not undergo any scheduled follow-up. However, participants who are NILM and HPV negative at 2 predetermined facilities, accounting for approximately 10% of the participants who are NILM and HPV negative, will receive a self-collection HPV testing kit 1 year after the baseline screening to evaluate the conversion rate of HPV positivity after childbirth.

At each prenatal checkup or at delivery, the participants will be asked to schedule their next visit to minimize loss to follow-up. Data obtained during pregnancy and up to the time of delivery will be retained and analyzed even for participants who discontinue the study or are lost to follow-up.

### Data Collection

The following data will be collected for each enrolled participant: study site, registration date, gestational age, participant’s age, gravidity and parity, history of abnormal cervical cytology and lesions, past screening history and results, cytology and HPV testing results, colposcopic findings, biopsy results, delivery outcome, gestational age at delivery, and mode of delivery.

In addition, questionnaire-based data will include marital status, history of oral contraceptive use, smoking history, HPV vaccination history, and questions about health literacy. Immunocytochemistry data from cervical swab specimens will also be collected.

### Safety Considerations

The trial interventions are limited to cervical scraping and colposcopy; cervical biopsy is not included because it is performed according to routine clinical practice criteria. To date, no severe adverse pregnancy outcomes related to cytology brushes have been reported [18]. Adverse events will be recorded if they meet the following criteria: grade 3 or higher according to the Common Terminology Criteria for Adverse Events [19], including miscarriage, grade 2 bleeding, or any unplanned visit requiring treatment. Only adverse events occurring within 1 week of the procedure will be collected given that potential effects of the intervention are expected to be short-lived. At each participating site, the investigators will assess the relationship of each adverse event to the study procedure and record the information in the electronic data capture form, and the prespecified statistical analysis will be performed after data lock. However, any unexpected or severe adverse events considered related to the study procedure will be promptly reported to the ethics committee.

### Sample Size and Statistical Analysis

All enrolled participants who undergo cervical sampling during early pregnancy and have evaluable baseline screening results will constitute the full analysis set. A per-protocol set, defined as participants in the full analysis set without major protocol deviations affecting assessment of the primary end point, will be used for sensitivity analyses. CIN2+ case detection via the HPV testing protocol is defined as the number of CIN2+ cases detected in participants with ASC-US or worse and who are HPV-positive at baseline screening. CIN2+ case detection via the cervical cytology protocol is defined as the number of CIN2+ cases detected in participants with ASC-US or worse and who are HPV-positive plus those with LSIL or worse and who are HPV-negative at baseline screening. Because the difference in the number of CIN2+ detections between each protocol corresponds to participants with LSIL or worse or who are HPV-negative, which is expected to be minimal, this trial is designed to demonstrate the noninferiority of the HPV testing protocol [15]. Table 1 summarizes the expected HPV positivity rate, the CIN2+ detection rate, and the estimated numbers of HPV-positive cases and CIN2+ detections among 5000 participants in this trial, based on previous literature.

**Table 1:** Expected proportions of each group classified by human papillomavirus (HPV) and cytology results and incidence of cervical intraepithelial neoplasia (CIN) 2 or worse.

Category			NILM^a^ and who are HPV-negative (%)		ASC-US^b^ and who are HPV-negative (%)		LSIL^c^ or worse and who are HPV-negative (%)			ASC-US and who are HPV-positive (%)		LSIL or worse and who are HPV-positive (%)			NILM and who are HPV-positive (%)
Proportion of each group in the overall population															
	Assumed proportion in this study	87.5		1		0.5			1		2			9	
	Range in previous studies [15,20,21]	85.1-89.4		0.7-1.2		0.7-1.2			0.6-1.4		0.7-2.9			7.4-10.8	
CIN2^+^ detection rate immediately after screening															
	Assumed proportion in this study	—^d^		—		8			13		26			—	
	Range in previous studies [15,22-25]	—		—		2.16-15.3			13-13.5		25.9-27.5			—	
	Estimated number per 5,000 participants	—		—		2			6.5		26			—	
Annual incidence of newly detected CIN2^+^															
	Assumed proportion in this study	—		0.3				—	3				—	1.5	
	Range in previous studies [23,24]	—		0.2-0.3				—	2.6-4.2				—	1.33-1.95	
	Estimated number per 5000 participants	—		0.2				—	4.5				—	6.8	

^a^NILM: negative for an intraepithelial lesion or malignancy.

^b^ASC-US: atypical squamous cells of undetermined significance.

^c^LSIL: low-grade squamous intraepithelial lesion.

^d^Not applicable.

The estimated detection rate of CIN2+ cases immediately after the baseline screening is 0.65% for the HPV testing protocol and 0.69% for the cervical cytology protocol [15,20-27]. Regarding the primary end point, the noninferiority margin was set at 0.132% assuming that HPV screening could detect an additional 0.132% of cases after 1 year. The target sample size was set at 5000 participants taking into consideration the possibility of accumulation. To ensure sufficient statistical power, the primary analysis will use a one-sided 10% noninferiority test using the method by Tango [28], and with 5000 cases, the study can achieve a statistical power of at least 80%. An interim analysis is not planned. The extent of missing data, including loss to follow-up, will be summarized descriptively. Study site characteristics will also be summarized, and their potential impact on the results will be considered as appropriate.

### Quality Assurance

Central monitoring will be conducted using data collected through an electronic data capture system. Queries will be generated as needed to ensure data accuracy and completeness. An audit will not be conducted because the study intervention is minimally invasive and considered low risk. Study conduct and data integrity will be ensured through centralized monitoring.

### Ethical Considerations

This trial was approved by the Ethics Committee for Life Science and Medical Research Involving Human Subjects at Yokohama City University on July 25, 2024 (approval F240704001), and the current protocol version is 1.2. The ethical and social acceptability of the study was reviewed by an institutional ethics committee that includes lay members, ensuring public oversight without direct patient and public involvement. The protocol and statistical analysis plan can be obtained from the study office at the Department of Obstetrics and Gynecology, Yokohama City University. Written informed consent will be obtained from all research participants who meet the eligibility criteria. Each participant will be assigned a unique study ID, anonymized data will be securely managed at the data center, and direct contact with research participants will be facilitated through a call center. Personal information will be used only for the identification of participants and for limited purposes, such as sending test kits and results or a gift card in compensation. This study may involve future secondary use of data and biological samples. Therefore, the participants will be informed of this possibility at the time of consent, and their agreement to such use will be confirmed. If the samples are to be used for additional research, a new research protocol will be prepared, which will be approved by the ethics committee, and an opt-out procedure will be implemented before use. A gift card valued at ¥500 (US $3.15) will be provided to participants as a token of appreciation for their participation. This study is registered in the Japan Registry of Clinical Trials under the clinical research implementation plan number jRCT1030240280, which was issued on February 26, 2025. Major changes to the protocol will be approved by the ethics committee, reported to the principal investigator, and reflected in the Japan Registry of Clinical Trials. The research plan and results will be published.

## Results

Funding for this study was provided in April 2024. The study protocol was approved by the ethics committee on July 25, 2024. Data collection commenced in September 2024 and is scheduled to be completed by December 2026. As of March 2026, a total of 1906 participants had been enrolled. Data analysis is currently planned to begin in December 2027, and the study findings are expected to be published in 2028.

## Discussion

In this study, the primary end point is to assess the noninferiority of HPV testing compared with cytology for detecting CIN2+ lesions during pregnancy. Secondary end points include evaluating whether participants who test negative during pregnancy remain negative post partum, thereby informing the appropriateness of screening intervals and examining the effect of false-positive results through events identified at 1 year of follow-up.

The NILM and HPV-positive group is eligible for 1-year follow-up only under the HPV testing protocol, whereas the ASC-US and HPV-negative group is eligible for 1-year follow-up only under the cytology screening protocol. However, in either group, if participants do not attend the 1-year follow-up visit, CIN2+ cases detected at that time may be underestimated. Importantly, these groups are not recommended to undergo additional diagnostic evaluation during the remainder of the pregnancy and, therefore, do not contribute to the CIN2+ detection rate during pregnancy. In this study, we focused on ensuring that missed CIN2+ cases under the HPV testing protocol are minimized in the unique clinical setting of pregnancy; therefore, the primary end point was defined as the noninferiority of the CIN2+ detection rate during pregnancy. On the basis of existing data, the HPV testing protocol is estimated to detect an additional 0.132% (6.6 of 5000) of CIN2+ cases within 1 year. Therefore, the noninferiority margin was set at 0.132%, assuming that a small number of cases not detected during pregnancy via the HPV testing protocol could be identified at follow-up. This margin is considered clinically acceptable in light of American Society for Colposcopy and Cervical Pathology guidance that immediate confirmatory evaluation is not required for populations with a risk of CIN grade 3 or higher of less than 0.4% [24]. Compared with the cytology protocol, the HPV testing protocol is expected to increase the number of individuals requiring repeat testing at 1 year by 8% (400/5000). In contrast, 87.5% (4375/5000) of the participants may be eligible for an extended screening interval from 2 to 5 years, which is expected to reduce the overall testing burden. Thus, if CIN2+ case detection during pregnancy via the HPV testing protocol is noninferior to that of the cytology protocol, introducing the HPV testing protocol would have substantial clinical value.

During pregnancy, the accuracy of cervical cytology may be compromised by increased mucus production and shifts in cervical position, and some cases of invasive cervical cancer missed through cytology have been reported. Although the comparable efficacy of cervical cytology between pregnant and nonpregnant cohorts has been described [29], baseline characteristics were not adjusted for. In contrast, HPV testing has demonstrated robust efficacy in the general population even when performed using vaginal swab samples rather than cervical swabs [30], suggesting that it is less affected by mucus production and cervical position. These findings indicate that HPV testing may be a reliable method for cervical cancer screening during pregnancy, with the additional potential benefit of extending screening intervals. However, the effectiveness of HPV testing has not been sufficiently validated.

The global HPV infection rate among pregnant women is an average of 30.8% [31], with considerable variation by region and age; in Japan, reported rates range from 6.1% to 32.5% [26,32]. Some studies have shown that HPV infection is more prevalent in pregnant women than in nonpregnant women of the same age group, which may be related to changes in immune status and sex hormone levels [33].

Validation in the pregnant population is essential because baseline characteristics may differ between the general and pregnant populations. Previous studies have indicated that HPV testing during pregnancy is feasible, with high lesion detection rates [34]. However, these studies did not use the HPV testing protocol that is currently used in many countries. Moreover, the required sample size and statistical power were not evaluated in advance.

To date, no statistically designed clinical trials have directly compared the effectiveness of HPV testing and cytology for cervical cancer screening during pregnancy. Trials require the enrollment of a large number of participants because cancer screening has an inherently low detection rate. Although large-scale prospective studies are often limited by financial and logistical challenges, this trial has secured sufficient funding and begun enrollment. The results of this trial will provide critical evidence to determine whether an HPV testing protocol should be adopted as the primary cervical cancer screening method in pregnant women because of its potential advantage of extended screening intervals or to determine whether the current cytology-based protocol should be maintained. However, this study has several limitations, including its single-arm design, short follow-up period, and the fact that biopsy decisions are based on real-world clinical practice and left to the discretion of the attending board-certified obstetrician-gynecologist. If a biopsy is not performed, this may introduce verification bias.

The findings of this study are expected to provide evidence to inform whether the HPV testing protocol should be introduced for pregnant women or whether the current cytology protocol approach should be maintained. The findings of this study will be widely disseminated through publication in peer-reviewed international journals and presentations at domestic and international academic conferences. In addition, as many institutions registered with the Japan Society of Obstetrics and Gynecology are participating in this study, we plan to use the knowledge gained from this study to advocate that the society develop cervical cancer screening guidelines for pregnant women.

## Data Availability

Deidentified data generated during this study will be made available after completion of the final analysis to the extent permitted by ethical restrictions.

## Appendix

Multimedia Appendix 1 List of participating facilities in this trial as of March 2026.

## Abbreviations

AGC: atypical glandular cells
AIS: adenocarcinoma in situ
ASC-H: atypical squamous cells cannot exclude a high-grade squamous intraepithelial lesion
ASC-US: atypical squamous cells of undetermined significance
CIN: cervical intraepithelial neoplasia
CIN2+: cervical intraepithelial neoplasia grade 2 or higher, adenocarcinoma in situ, and invasive cervical cancer
HPV: human papillomavirus
HSIL: high-grade squamous intraepithelial lesion
LSIL: low-grade squamous intraepithelial lesion
NILM: negative for an intraepithelial lesion or malignancy
SCC: squamous cell carcinoma
