# Supplementary material for: Evaluation of the Efficacy of Human Papillomavirus Screening Compared With Cytology Screening in Pregnant Women: Protocol for a Prospective Multicenter Trial
Source: JMIR Res Protoc. 2026 Jun 25;15:e86397. doi: 10.2196/86397 (PMC13351641; doi:10.2196/86397)
Supplement: Multimedia Appendix 1 [file resprot_v15i1e86397_app1.docx]

Table S1: List of participating facilities in this trial as of 9 March 2025

| Yokohama City University School of Medicine, Yokohama, Kanagawa, Japan |
| --- |
| Yokohama City University Medical Center, Yokohama, Kanagawa, Japan |
| Yokosuka Kyosai Hospital, Yokosuka, Kanagawa, Japan |
| Saiseikai Yokohamashi Nanbu Hospital, Yokohama, Kanagawa, Japan |
| National Hospital Organization Yokohama Medical Center, Yokohama, Kanagawa, Japan |
| Yokohama Rosai Hospital, Yokohama, Kanagawa, Japan |
| Yokohama Municipal Citizens’ Hospital, Yokohama, Kanagawa, Japan |
| Yokohama Minami Kyosai Hospital, Yokohama, Kanagawa, Japan |
| Odawara Municipal Hospital, Odawara, Kanagawa, Japan |
| Fujisawa City Hospital, Fujisawa, Kanagawa, Japan |
| Yamato Municipal Hospital, Yamato, Kanagawa, Japan |
| Keiyu Hospital, Yokohama, Kanagawa, Japan |
| Teikyo University Hospital, Itabashi, Tokyo, Japan |
| Nippon Medical School Musashikosugi Hospital, Kawasaki, Japan |
| Matsue City Hospital, Matsue, Shimane, Japan |
| Saiseikai Yokohamashi Tobu Hospital, Yokohama, Kanagawa, Japan |
| Koshimizu Obstetrics and Gynecology Clinic, Hiratsuka, Kanagawa, Japan |
| Hana Obstetrics and Gynecology Clinic, Yokohama, Kanagawa, Japan |
| Niigata University Medical & Dental Hospital, Niigata, Niigata, Japan |
| Yokohama City Minato Red Cross Hospital, Yokohama, Kanagawa, Japan |
| National Hospital Organization Tokyo Medical Center, Meguro, Tokyo, Japan |
| Kanto Rosai Hospital, Kawasaki, Kanagawa, Japan |
| Kumamoto University Hospital, Kumamoto, Kumamoto, Japan |
| Nagasaki University Hospital, Nagasaki, Nagasaki, Japan |
| Sui Obstetrics & Gynecology, Yokohama, Kanagawa, Japan |
| Shiga University of Medical Science Hospital, Otsu, Shiga, Japan |
| Sapporo Medical University Hospital, Sapporo, Hokkaido, Japan |
| Social Welfare Corporation Boshi Aiiku Kai Comprehensive Maternal and Child Health Center, Tokyo, Japan |
| Kannai Lady’s Clinic, Yokohama, Kanagawa, Japan |
| Tsuzuki Lady’s Clinic, Yokohama, Kanagawa, Japan |
| Osada Obstetrics and Gynecology Clinic, Kofu, Yamanashi, Japan |
| Shimizu Obstetrics and Gynecology Clinic, Kofu, Yamanashi, Japan |
| Hiroshima University Hospital, Hiroshima, Hiroshima, Japan |
| Kanako Ladies Clinic Sugita, Yokohama, Kanagawa, Japan |
| Kanako Ladies Clinic Yokohama Minatomirai, Yokohama, Kanagawa, Japan |
